# Multiple Comminuted Fracture of the Sesamoid Bones of Both Forward Extremities

**Published:** 1883-07

**Authors:** 


### X.—MULTIPLE COMMINUTED FRACTURE OF THE SESAMOID BONES OF BOTH FORWARD EXTREMITIES.

REPORTED FROM DR. McLELLAN'S CLINIC.

This case has been abstracted from the Clinical Reports of the Columbia College for the Winter of 1882–3.

*History of Case.*

The animal from which the specimens were obtained was a car horse which was taken from the stable in the morning apparently sound. After being driven for a short distance he slipped; but according to the driver's statement, did not fall for some time. The driver at once saw that the animal was too severely injured to finish the trip, and he was at once returned to the hospital.

He was brought into the hospital of the car stable just as the clinic was in progress, and was there examined by Dr. McLellan and a large number of students from the college.

When compelled to move he would carry his posterior extremities well forward, the anterior portion of the body straight, and positively refuse to take step by step.

The animal when brought out upon the floor manifested symptoms of great agony. Then he would lie down, which seemed to give him great relief. The anterior extremities were in a state of semi-flexion.

With great exertion and considerable assistance the horse managed to regain his feet unaided, but upon standing a very few minutes evinced great suffering, which apparently increased with every extra minute that he stood.

The symptoms were similar to those of acute laminitis, and might, without careful manipulation, be mistaken for that disease.

The history in a case like this would rather bar out laminitis. The feet of the animal, standing well back upon his heels, with joints to the knee in semi-flexion, would indicate something further than laminitis.

The parts were manipulated and examined as well as could be without securing the animal, and a hasty diagnosis made.

This was rupture of the flexor pedis perforans tendon, near the fetlock.

The prognosis rendered was unfavorable, and the animal was at once destroyed.

*Necropsy*—Immediately. As there was no indication of internal disease, none of the viscera were removed. The limbs were removed and taken to the college dissecting rooms for closer study.

[When the joint was examined distinct crepitation could easily be detected, and there ought not to have been any difficulty in detecting it during life, as there apparently had been no great swelling prior to death.

The way in which the injury occurred we think quite easy of explanation. The posterior extremities shot forward under the body, causing him to sit suddenly down. This would have a tendency to cause the anterior extremities to shoot forward and let the weight of the body down upon these sesamoid bones. The efforts of the animal to rise, and the pounding of the bones upon the irregular paving stones, would easily explain the cause of the injury.

We have often seen animals struggling in this awkward position, and wondered why they were not more severely injured. Treatment, of course, would be of little avail, unless well supported by an immovable apparatus. —EDS.]

